# Migrants in transit through Mexico to the US: Experiences with violence and related factors, 2009-2015

**DOI:** 10.1371/journal.pone.0220775

**Published:** 2019-08-21

**Authors:** René Leyva-Flores, Cesar Infante, Juan Pablo Gutierrez, Frida Quintino-Perez, MariaJose Gómez-Saldivar, Cristian Torres-Robles

**Affiliations:** 1 Health Systems Research Center, National Institute of Public Health, Cuernavaca, Morelos, México; 2 Center for Policy, Population and Health Research, School of Medicine, National Autonomous University of Mexico, Mexico City, Mexico; 3 College of Science and Humanities, Autonomous University of Mexico City, Mexico City, Mexico; 4 Faculty of Medicine, University of Valparaiso, Valparaiso, San Felipe, Chile; Harvard Medical School, UNITED STATES

## Abstract

**Objectives:**

The objectives of the study are to 1) estimate the burden of physical, sexual, and psychological violence among migrants in transit through Mexico to the US; and 2) examine the associations between experiencing violence and sociodemographic characteristics, migratory background, and health status in this vulnerable population.

**Method:**

A cross-sectional study combining qualitative and quantitative methods was carried out from 2009 to 2015 with a sample of 12,023 migrants in transit through Mexico to the US. Information on gender (male, female, and transsexual, transgender and transvestite -TTTs-); nationality; health status; migratory background; and experiences with violence was obtained. Fifty-eight migrants participated in in-depth interviews to explore any experiences of violence during their journey. A descriptive analysis was performed and a probit regression model was applied to analyze the factors associated with violence. Qualitative information was analyzed to understand experiences, meanings and responses to violence.

**Results:**

The overall prevalence of suffering from any form of violence was 29.4%. Nearly 24% reported physical violence, 19.5% experienced psychological violence, and approximately 2% reported sexual violence. TTTs experienced a significantly greater burden of violence compared to men and women. Violence occurred more frequently among migrants from Central American (30.6%) and other countries (40.0%) than it did among Mexican migrants (20.5%). Experiences involving sexual, physical and psychological violence as well as theft and even kidnapping were described by interviewees. Migrants mistrust the police, migration authorities, and armed forces, and therefore commonly refrain from revealing their experiences.

**Conclusion:**

Migrants are subjected to a high level of violence while in transit to the US. Those traveling under irregular migratory conditions are targets of even greater violence, a condition exacerbated by gender inequality. Migrants transiting through Mexico from Central American and other countries undergo violence more frequently than do Mexican migrants. Protective measures are urgently needed to ensure the human rights of these populations.

## Introduction

Throughout the world, traditional migration routes have turned into high-risk corridors with alarming consequences for the safety, integrity and health of migrants [1,2,3,4,5,6,7].

In an effort to understand the vulnerability of migrants, various studies have explored related socioeconomic factors, political issues and migration policies in both countries of origin and destination [8, 9, 10, 11,12].

Globally, there were an estimated 258 million international immigrants in 2017 of which 57% have established themselves in developed regions. Among this group, 61% originated from developing countries [13]. The largest number of immigrants resided in the United States (US), which hosted 49.8 million (19% of the world’s total) in 2017. The migration corridor from Latin America and the Caribbean to Northern America was the third largest worldwide in 2017, with over 26 million international migrants [13]. For Mexico and Central American countries, the US is the primary country of destination [13, 14]. The World Bank (2017) estimates the number of migrants from Mexico and Central American countries in the US as follows: Belize (48,918), Costa Rica (85,133), El Salvador (1,387,022), Guatemala (935,707), Honduras (651,059), Mexico (11,573,680), Nicaragua (243,024) and Panama (94,958) [13]. In regards to undocumented migrants from Central America entering Mexico, the Mexican Ministry of the Interior estimated 390,000 individuals in 2014 [15].

Migrants are frequently subjected to violence in their home countries and continue to be victims of violence throughout their transit in Mexico, while crossing the US border and at their destination, where they often remain with an irregular migratory status [16,17]. Between 1998 and 2015 a total of 6,571 migrants were found dead in the US and in 2015 the US-Mexico border ranked third globally with the highest number of migrants dead and missing [18]. Furthermore, Mexico is a major transit country for Central American migrants travelling to the US; many go missing on the journey, and many unidentified human remains are found [19]. Also, according to the National Human Rights Commission in Mexico, migrants in transit are prey to varied forms of violence; despite this danger, 88% of the victims decide to continue their travel [7]. Those that succeed in entering the US live in constant fear of being deported. It is estimated that, overall, during the Obama administration about 3 million immigrants were deported between 2009 and 2017 [17,20]. According to US Immigration and Customs Enforcement, during the Trump administration, removals increased by 17% in 2017, and in the first semester of 2018 a total of 109,296 repatriation events of Mexicans were registered [21].

In this social context, non-governmental human rights organizations have come forward to report and document cases of extreme violence [22]. However, few studies have addressed both the magnitude and forms of violence perpetrated or the personal experiences of the victims as they grapple with violence in Mexico.

The twofold purpose of this study was to: estimate the overall burden of violence, including physical, sexual, and psychological violence, experienced by migrants in transit through Mexico to the US from 2009 to 2015; and to examine factors placing people at risk of violence in this vulnerable population.

## Methods

This paper is part of a broader research project initiated in 2002 in collaboration with migrant shelters in Mexico (*Casas del migrante*). These faith-based non-governmental organizations not only provide humanitarian services; they also promote and defend the human rights of migrants. A cross-sectional study that combined quantitative and qualitative methods was carried out between February 2009 and December 2015 in five *Casas del migrante* located at strategic points along the migrant transit route. The shelters were located in the cities of Tapachula in the state of Chiapas; Ixtepec in Oaxaca; San Luis Potosí (SLP); Saltillo in Coahuila; and Tijuana in Baja California Norte. All of these sites are located along the railway of a cargo train, which to date, provides one of the most important means of transportation for irregular migrants in Mexico [7].

During this period, a strategy to promote human rights, facilitate access to health care, prevent HIV/AIDS, and alleviate sexual and reproductive health problems, was implemented in the *Casas del migrante*. At these shelters, group activities were carried out with migrants in order to provide them with basic information on the subjects previously mentioned. After the activities, migrants were invited to participate voluntarily, in a non-random and confidential manner, to answer a questionnaire that gathered information on their sociodemographic characteristics, gender, migratory background, country of origin, and experience of different risks and types of violence during their transit through Mexico. Staff from the shelters that were trained by the research team administered the questionnaire. The training lasted three days whereby researchers and staff from the shelters reviewed the objectives of the study, the importance of the different variables included in the questionnaire and ethical issues related to data collection. Subsequently, researchers supervised the collection of the data and provided feedback to the staff. Information was also gathered regarding sexual behavior during the trip, specifically focusing on casual sex, condom use at last sexual relationship, and relations under the influence of alcohol or drugs. Finally, those who indicated having experienced violence were asked whether or not they had reported the event to the authorities or to human rights civil society organizations. A total of 12,023 migrants participated voluntarily and their informed consent was obtained: 3,941 at Tapachula, 1,861 at Ixtepec, 2,503 at SLP, 2,334 at Saltillo, and 1,384 at Tijuana. *Casas del migrante* did not gather data on non-participants, but reported that there were few of those invited that declined participation.

This study combined qualitative and quantitative methods using a complementary approach. In such a design, qualitative and quantitative approaches are used to examine overlapping but also different facets of a certain phenomenon seeking for an enriched, elaborated understanding. This allowed us to describe the sociodemographic characteristics of migrants, provide an estimate of the frequency of self-reported violence, as well as further explore and understand the violent experiences migrants have faced while in their transit through Mexico [23]. First, quantitative data were collected through the questionnaire following the procedures described above. This strategy allowed us to identify migrants that experienced violence who were then invited for an in-depth interview. For both quantitative and qualitative analysis, violence was the main consideration for analysis, and it was defined as any action perpetrated by a person or group causing direct harm to another person and generally occurring in circumstances of unequal power relations [24,25,26]. The dimensions of violence included in this analysis were: a) Physical violence (beatings, thefts, extortions and kidnappings); b) Psychological violence (humiliation, threats, rejection, insults); and c) Sexual violence (rape or sex in exchange for goods, money, protection, transportation and/or food, among others) [24,25,26]. In order to maximize available quantitative information, we used data available for each variable, i.e. the number of respondents was different for some variables; this was in order to avoid discarding observations due to periodic adjustments to the questionnaire used in this survey from 2009 to 2015. Missing values were excluded from the analysis.

We recognize that our categories generalized the experiences of migrants by highlighting a particular characteristic of violence according to the perception of the victim. It is a fact, however, that violent events often include various forms of violence simultaneously [7,24,25,27,28]. For the quantitative data, descriptive statistics were reported in order to compare the characteristics of the populations affected and not affected by violence, while pinpointing gender-based violence experiences. Point estimates for the differences by experience of violence and by gender were calculated. Finally, a probit regression model was used to analyze the variables associated with the violent experiences of migrants (outcome variable), and to identify the marginal effects of these variables. For these models, the dependent variable is any experience of violence as reported by migrants and is coded as “yes” or “no.” Independent variables are gender, schooling, country of origin, migration experience, having children, experience of discrimination and year of the survey. In the probit model, the coefficients aim to estimate the probability of a given observation to fall in a particular category, which in this case is the outcome of experiencing violence during transit.

Design effect in data analysis was considered including each *Casa del migrante* as a primary sampling unit, and each year as a stratum [29]. This allowed adjustment of estimates both for similarities among the populations served by each *Casa del migrante* and for changes in the variables of interest over time. This design effect is included both in descriptive and probit analysis. Statistical analysis was performed using Stata, version 15.0.

### Qualitative component

In-depth interviews were held with migrants who reported in the questionnaire some form of violence during their transit through Mexico. Informants were included according to the following criteria: having experienced violence (sexual, psychological or physical), gender, and country of origin. Following these criteria 30 men and 28 women were interviewed, with a mean age of 30.7 years, from Guatemala, El Salvador, and Honduras. The interviews were conducted by two researchers and were held in private spaces within the shelters in order to guarantee confidentiality and privacy for the informants. Interviews were audio recorded and lasted between 45 and 60 minutes each.

The objective of the qualitative component was to understand the meaning of the violence experienced by migrants. The codes were based on the existing literature that explores violence and previous research undertaken by the research team [7,24,25,27,28]. An interpretivist approach to data analysis was adopted in which emphasis was placed on the individual’s experience of migration, the different forms of violence and its consequences [30]. Initial analysis took place during data collection by listening to recordings, transcribing, and making field notes. As a result of this process, the different dimensions of violence (psychological, physical and sexual) present in the migrants’ discourse could be identified. To enhance reliability, members of the research team continuously discussed the coding scheme, the development of themes and categories, and the interpretation of the data [30]. Atlas.ti computer software was used to organize and analyze the qualitative data.

Participants were advised of the purpose of the interviews and asked to provide their informed consent to being audio-recorded. The confidentiality of respondents was guaranteed by assigning a code to each interview. Procedures for contacting migrants, conducting interviews and surveys, processing data and publishing related information were approved and monitored by the Ethics Committee of the National Institute of Public Health in Mexico (Registration Code 917).

## Results

The sociodemographic characteristics, migratory background and health status of the migrants who did/did not suffer violence while in transit through Mexico and provided data for the quantitative analysis are presented in Table 1.

**Table 1:** Migrants in transit through Mexico: Sociodemographic characteristics, migratory background and health status according to experience with violence, 2009–2015.

		Total population	Violence		
		n = 12,023	n = 8,484	n = 3,539	
	Variable	Percentage (95% confidence interval)	No Percentage (95% confidence interval)	Yes Percentage (95% confidence interval)	p-value*
Gender	Male	77.72	76.10	81.60	0.074
n = 12,023		(65.98–89.46)	(63.81–88.38)	(70.44–92.77)	
	Female	21.73	23.55	17.35	0.048
		(9.96–33.49)	(11.28–35.82)	(6.11–28.59)	
	Transexual, transgender, transvestite	0.56	0.35	1.05	0.005
		(0.24–0.87)	(0.10–0.61)	(0.51–1.58)	
Marital status	Has partner	42.86	43.35	41.68	0.16
n = 11,824		(40.00–45.81)	(40.69–46.01)	(37.60–45.76)	
Education	Years of schooling	7.04	6.96	7.24	0.013
n = 10,940		(6.76–7.32)	(6.67–7.25)	(6.93–7.54)	
Has children	Yes	66.68	66.63	66.78	0.887
n = 10,815		(63.17–70.18)	(63.09–70.18)	(62.89–70.67)	
	No	2.43	2.47	2.32	0.02
		(2.29–2.57)	(2.32–2.62)	(2.18–2.47)	
Country of origin	Mexico	12.01	13.53	8.36	0.15
n = 12,023		(0.15–23.87)	(0.67–26.39)	(2.30–19.03)	
	Honduras/Guatemala/El Salvador	81.92	80.53	85.25	0.182
		(70.78–93.06)	(68.46–92.60)	(75.06–95.44)	
	Others	6.07	5.94	6.39	0.435
		(4.95–7.19)	(4.70–7.19)	(5.17–7.60)	
Migratory background	Number of attempts to reach US	2.40	2.34	2.53	0.022
n = 11,425		(2.27–2.53)	(2.21–2.47)	(2.35–2.71)	
	Has reached US	43.24	42.70	44.50	0.429
		(37.80–48.68)	(37.22–48.18)	(37.86–51.14)	
	Has stayed in another *Casa del migrante*	67.53	64.46	75.05	0.026
		(57.44–77.63)	(53.43–75.49)	(65.02–85.08)	
Health status	Has had health problem	39.27	36.50	45.92	0.053
n = 12,023		(31.06–47.49)	(26.04–46.97)	(39.18–52.65)	
Sexual behavior during trip	Has had sex	16.70	14.32	22.30	0.001
		(13.13–20.26)	(10.49–18.14)	(17.64–26.96)	
n = 10,470	Used condom at last sex	43.32	43.95	42.09	0.571
		(35.33–51.31)	(34.18–53.71)	(36.73–47.45)	
	Has had sex under the influence of alcohol or drugs	19.95	16.22	25.59	0.001
		(14.86–25.04)	(11.62–20.81)	(19.61–31.56)	

*p <0.05 comparison of groups who did/not experience violence

The number of observations presents differences among variables as a result of missing values.

Source, Leyva R, Infante C. Multicenter Project: International Migration and Sexual and Reproductive Rights of Migrants from Mexico and Central America, 2009–2015.

As reported, 3,539 of the total 12,023 migrants experienced violence: that is, 29.4%. Prevalence of violence was 30.9% for males, 23.5% for females and 55.2% for TTTs. Reporting of violence by location of *Casa de migrante* ranged from 18.3% in Tapachula to 38.3% in Saltillo.

The majority of migrants came from Central America (81.9%) and had an average of seven years of schooling. Up to 42.8% reported having an intimate partner and two thirds (66.8%) had children. In regards to their migratory experience, the number of previous attempts to enter the US was 2.4 on average, and 43.2% had reached the US. In relation to health, 39.2% reported a health problem or an accident in the two weeks prior to the interview, 16.7% mentioned having sexual relations during their current transit, and violence was reported by 29.4% of migrants.

Statistically significant differences were observed by gender (p-value for the difference in the proportion of experience violence between males and females is 0.053, males and TTTs 0.003, and between females and TTTs 0.001 –data not shown in the table), education, health status (marginally significant with p-value of 0.053) and frequency of sexual relations during the trip between migrants who reported having suffered and those who did not report some kind of violence. Additionally, of the total number of migrants who reported having had sexual relations during the trip, violence occurred most frequently among those who did so under the influence of alcohol or drugs (Table 1).

Overall, 19.5% of all migrants reported psychological violence, 23.7% physical violence, and 1.6% sexual violence. Of those that reported suffering violence, psychological violence was reported more frequently by women and TTTs (52.7% and 64.8%, respectively) in comparison with men. Humiliation was the most common form of psychological violence affecting women and TTTs (52.7% and 64.8%, respectively) and threat was the most common form of psychological violence affecting males (55.9%).

Rejection for being female was reported by 9.19% of women. Only 1.3% of all migrants felt rejected for being indigenous. Sexual preference was reported as motive of rejection by 1.0% of all migrants, although for TTTs this was reported by 47.7%. Being undocumented was reported as motive of rejection by 25.9% of all migrants, and was higher for females and TTTs as compared to males (46.7%, 32.8% and 20.0%, respectively, with p-values of 0.008 for the difference between females and males and no significance for other differences). Rejection and discrimination by Mexicans against migrants in transit stem from common stereotypes regarding the undocumented, including stigmatizing labels such as “wetbacks” and “tattooed,” which dismiss the migrants as delinquents or violent gang members.

“The local people treat us the same. It doesn’t matter if you are from Honduras or Nicaragua because we are wetbacks, we don’t have documents to migrate. People have discriminated [against] me because I have tattoos. I want to have a better life and that’s the reason I am here away from my family in El Salvador. I am asking for refugee [status] in Mexico but it is difficult to have a relationship with other persons because of my tattoos. There was a time when I did bad things but I am a human being and I am uncomfortable of being discriminated against just because I am from abroad.” (Male migrant from El Salvador, 33 years of age)

Given the situations of violence in their home communities, some migrants, including the ones currently interviewed, seek refugee status or asylum in Mexico. Nonetheless, as is shown in the previous testimony, they tend to experience and perceive great difficulties in gaining this migratory status. Regarding those that experienced physical violence, extortion and theft of belongings ranked as the most common (68.7%), followed by beating (24.1%) and kidnapping (9.6%). No significant differences were observed according to gender (Table 2).

**Table 2:** Migrants in transit through Mexico: Forms of violence by gender, 2009–2015.

	Variables	General N = 12,023 Percentage (95% confidence interval)	Male N = 9,344 Percentage (95% confidence interval)	Female N = 2,612 Percentage (95% confidence interval)	TTTs N = 67 Percentage (95% confidence interval)	p-value (1)	p-value (diff male/TTTs)	p-value (diff fem/TTTs)
Suffered some form of violence		29.44	30.91	23.51	55.22	0.053	0.003	0.001
n = 12,023		(22.57–36.30)	(23.61–38.20)	(16.00–31.01)	(40.54–69.91)			
Psychological violence	Humiliation	37.92	34.42	52.77	64.86	0.037	0.001	0.295
n = 3,539		(28.61–47.24)	(25.14–43.69)	(35.60–69.94)	(46.68–83.05)			
	Threat	52.30	55.99	35.34	45.95	0.015	0.17	0.356
		(40.39–64.21)	(44.50–67.49)	(18.24–52.45)	(25.75–66.14)			
Rejection:	for being a woman			9.19				
n = 11,225				(6.47–11.91)				
	for being indigenous	1.38	0.97	2.72	1.49	0.000	0.689	0.365
		(0.87–1.89)	(0.62–1.32)	(1.84–3.60)	(-1.14–4.13)			
	because of sexual preference	1.09	0.76	1.07	47.76	0.304	0.000	0.000
		(0.69–1.49)	(0.41–1.11)	(0.58–1.56)	(31.83–63.69)			
	because of undocumented status	25.90	20.02	46.75	32.84	0.008	0.141	0.245
		(16.94–34.86)	(13.79–26.26)	(27.03–66.46)	(17.30–48.37)			
	for not having money	12.40	11.94	13.63	28.36	0.489	0.039	0.055
		(8.62–16.19)	(7.68–16.21)	(9.01–18.25)	(14.57–42.15)			
	because of age	2.42	2.17	3.33	1.49	0.192	0.616	0.276
		(1.53–3.31)	(1.23–3.12)	(1.66–5.00)	(-1.14–4.13)			
Physical violence	Kidnapping	9.66	9.38	10.75	13.51	0.61	0.406	0.58
n = 3,539		(5.58–13.75)	(4.87–13.89)	(5.72–15.78)	(3.13–23.89)			
	Theft	68.72	71.85	53.75	72.97	0.052	0.918	0.164
		(53.73–83.71)	(56.18–87.52)	(35.29–72.20)	(52.37–93.57)			
	Beating	24.16	24.48	22.64	24.32	0.524	0.982	0.82
		(19.54–28.78)	(19.55–29.41)	(16.37–28.91)	(10.78–37.87)			
Sexual violence	Rape	3.90	1.49	14.17	21.62	0.000	0.003	0.243
n = 3,539		(2.13–5.67)	(0.73–2.24)	(10.19–18.15)	(9.14–34.10)			
	Sex in exchange for goods and/or money	2.63	1.97	4.07	29.73	0.025	0.006	0.01
		(1.47–3.78)	(0.78–3.16)	(2.45–5.70)	(10.64–48.82)			

(1) for difference between males and females

The number of observations presents differences among variables as a result of missing values.

Source, Leyva R, Infante C. Multicenter Project: International Migration and Sexual and Reproductive Rights of Migrants from Mexico and Central America, 2009–2015

Theft of personal belongings is one of the forms of violence making the transit through Mexico even more precarious.

“Some local police guys came, searched us and took 1,500 pesos (US 87.1) from us. They said: ‘this is fine. If not, we would have handed you over to Immigration’.” (Migrant, male, 20 years of age, from Guatemala)

In addition to theft of money and belongings as well as physical assault, aggressors commonly threaten to turn migrants in to authorities for deportation, as described in the following testimonies.

“He hit us with a club and they broke my rib. They wanted my money. If not, they were gonna throw her [friend] off [the train] and call immigration authorities. It wasn’t much; it was 300 pesos (US 17.5). I didn’t cower though. I pounced on one of them and he bit my finger and beat me with a stick. I gave him the money, and that’s how I got to Monterrey, all beat up.” (Migrants, female, 26 and 27 years of age, from El Salvador)

The theft, assaults, and threats finally amount to a form of “payment” by the migrants to the aggressors so that they may continue their journey or access the cargo train—the most utilized mode of transport across Mexico to reach the US.

One of the most serious manifestations of violence during transit is kidnapping of migrants by delinquent groups in Mexico, which in the most extreme cases can lead to the death of the victim when ransom is not paid. These events can occur individually or towards groups, and accounts of migrant kidnappings are frequently reported to other migrants during their transit, as shown in the following testimony:

“Some men got on [the bus]. One of them didn’t, and the driver stood with him, on the ground, laughing. They got all of us off and took us to a hotel. There, they searched us. Then a cab came for us and took us to a house. We got to the house and there they had another 30 people kidnapped: migrants, Mexicans, small kids.” (Migrant, TTT, 23 years of age, from Honduras)

In the same testimony, the complexity of the criminal network is noted, which can include drivers of public transport, owners of hotels, and more. Neither nationality nor age appears to be a factor in kidnapping of migrants in Mexico.

Of the total number of migrants having suffered some form of violence, 6.5% reported sexual violence: six out of ten were raped, and four out of ten performed sexual favors in exchange for goods (e.g., food, transportation, or clothing, and/or money). Sexual violence was observed more frequently among TTTs and women compared with men. 21.6% and 14.1% of females and TTTs reported rape, respectively, compared to only 1.5% of males (p-values of 0.000 and 0.003). Transactional sex was more common among TTTs and women,–but particularly in TTTs- (29.7% and 4.0%) compared to 2.0% for males (Table 2) (p-values of 0.006 and 0.025). The reported frequency of sexual violence shows a marked difference by gender. Experiences of sexual violence emerge as part of the unequal dynamic between migrants and the local population during their journey, and tend to occur in certain transit routes where there is significant presence of delinquent groups: in the train, in other modes of transportation, and other spaces.

“Only once, a person raped me right after I got on the train. (…) It was one of those workers that are there; on the railway (…) He grabbed me by the hair, got me off the train and put me in his car.” (Migrant, female, 35 years of age, from El Salvador)

“They said, ‘the deadline [for paying ransom] is over and we’re gonna screw you.’ They took off my pants and stripped me. He tried and I resisted. They couldn’t penetrate me completely and they made me bleed.” (Migrant, male, 51 years of age, from El Salvador)

The conditions of violence as described during transit have normalized sexual violence from a perspective of gender differentiation. In the cases of women, some perceive unwanted sexual relations–that were associated with violence, or in unequal and disadvantaged situations–as one of the tools necessary to facilitate their passage and protect themselves from greater harm.

“The advantage of being a woman is that men will help you just to have sex with you. Just think of it as paying for protection with [your] body.” (Migrant, female, 28 years of age, from Guatemala)

In the same context of inequality and violence, the threshold to accept sexual relations is lowered as part of the exchange necessary to ensure survival within the migration process.

“If we have to get things with sex then we have to do it. Maybe you don’t want to but they take advantage of it. But if I have to eat, wear a jacket or sleep somewhere, so I get it because there is no choice. You do everything on this trip. You have to drink water in puddles from the watering holes where cows have been. It’s what you do to survive.” (22 year old Gay man from Guatemala)

Food, clothing, water, and shelter for the night or to rest, are all human necessities that aren’t easily found in the insecure and precarious situations in which migrants find themselves. The presence of each of these elements differentiates the individual capacities of migrants to manage the aggression and violence they experience during transit. Even so, it is gender and nationality which weigh most heavily in determining the likelihood that a migrant will suffer violence while crossing Mexico, as seen in the following multivariable model.

Table 3 presents the results of the regression model designed to identify the factors related to suffering violence in any form. The multivariate analysis confirmed the relevance of gender in the likelihood of suffering violence: compared to males, TTTs proved 21.7 percent more likely and women 4.5 percent less likely than men to experience violence (model 1); however, after including demographic and migratory background variables (model 3), the TTTs’ differential dropped to 14 percentage points and the correlation was weak (p<0.1) while the females´ differential increased to 7.4 points (p<0.001).

**Table 3:** Marginal effects of factors associated with any experience of violence among migrants in transit through Mexico to US, 2009–2015.

		(1)	(2)	(3)
	Variables	Violence Marginal effects (standard error)	Violence Marginal effects (standard error)	Violence Marginal effects (standard error)
Sex	Male		-	-
	Female	-0.045*	0.075***	0.074***
		(0.025)	(0.024)	(0.021)
	TTTs	0.217***	0.130	0.140*
		(0.069)	(0.080)	(0.074)
Education	Years of schooling	0.001	0.001	0.001
		(0.001)	(0.001)	(0.001)
Country of origin	Mexico			
	Honduras/Guatemala/El Salvador	0.072	0.158***	0.171***
		(0.070)	(0.050)	(0.044)
	Others	0.071	0.166***	0.178***
		(0.064)	(0.047)	(0.041)
Has children	No			
	Yes		0.016*	0.018
			(0.009)	(0.011)
Has reached US previously	No			
	Yes		0.019	0.013
			(0.018)	(0.015)
Discrimination	No			
	Yes		0.249***	0.241***
			(0.024)	(0.021)
Year of survey	2011			0.056
				(0.076)
	2012			-0.068
				(0.082)
	2013			-0.047
				(0.070)
	2014			-0.072
				(0.095)
	2015			0.034
				(0.093)
	Observations	11,424	9,904	9,904

*p<0.1

** p<0.05

***p<0.01

Source, Leyva R, Infante C. Multicenter Project: International Migration and Sexual and Reproductive Rights of Migrants from Mexico and Central America, 2009–2015

Regarding country of origin, migrants from Central America and other countries were more likely to suffer violence than Mexican migrants (30.6% and 40.0% vs 20.5%). Finally, years of schooling, having children (only marginally significant in model 2) and having entered the US previously did not correlate with the likelihood of experiencing violence.

It is also important to highlight that migrants have minimal access to legal, health and other public services and little possibility of exercising their rights. Of the total number of migrants who suffered any type of violence during transit, only 13.9% reported the event to authorities or social organizations. Migrants experience various forms of violence throughout their journey to the US, carried out by different perpetrators that vary from the local population, the army, police and organized crime. The violence is so frequent and generalized that migrants have accepted it as part of the price they have to pay for migrating. The following statement conveys the perspective of the directors of the *Casas del migrante* regarding the violence suffered by migrants as they transit through Mexico: “In Central America and Mexico, violence is generalized and unpredictable. In the past, people knew where violent events were likely to happen… Now it is impossible to know where violence will occur… They carry their caskets on their backs from the moment they leave home, because any day, any place, they are apt to encounter death”.

## Discussion

Globally, the dynamics of population mobility are growing complex, and human rights violations are frequent and devastating. Risk exposure under migratory conditions is escalating, and the effects of these factors on health are mirrored in increasingly acute problems, or even death [7,18]. Further aggravating the situation is the framework of restrictive measures and policies surrounding migration [19,20,21,31,32].

Violence against migrants starts in their own countries of origin and persists throughout their transit in Mexico and into their destinations [7,16,17,27]. Under these precarious conditions, violence is neither unknown nor unexpected to migrants. However, leaving everything behind to flee from violence in their home communities does not necessarily lead migrants to a secure place. Most are aware of the distinctively violent social conditions they must contend with in Mexico [6,33,34]. And once in their destination of the US, the majority of those who succeed in crossing the border are constantly haunted by the fear of being deported [20,21,35].

The results of our study suggest that migrants from Central American and other countries are more likely to experience violence while in transit to the US than are Mexican migrants. This may be attributable to their irregular migratory conditions, minimal social support, and certainly the violent conditions encountered in Mexico [7,19,24,25,27,28,34]. The majority of these migrants seek to evade, rather than face, the myriad risks inherent in their journey, namely the presence of migration authorities, organized crime and common criminals. Migrants from Central American and other countries prefer to take “safer” alternate routes: ones less controlled by authorities but, by the same token, are teeming with criminal organizations [19,34].

In order to understand the dimensions of the violence narrated by the migrants in transit through Mexico, we used the experiences of the local Mexican population as reference, and found that they were not significantly different. Over all migrants interviewed in this study, 29.4% reported having experienced some form of violence, versus. 28.2% of the local Mexican population [35]. There exists a widespread distrust of authorities, which, in the case of transitory migrants, prevailed among travelers from Central America and other countries who feared deportation [36,37]. Of the total number of migrants who have suffered some form of violence, only one out of ten reported the event to an authority or a human rights organization. Similarly, only 10.7% of Mexicans reported their violent experiences to the authorities [35].

Regarding the distribution of violence by gender, we identified that, overall, men suffered more violence than women, but less than TTTs. In contrast, within the Mexican migrant population, we found no statistically significant variation in the distribution of violence between male and female victims [35]. However, in our study, the distribution of violence according to gender shows that TTTs experience the highest incidence of all forms of violence. The number of TTTs included in the study, only 67, is a limitation in drawing conclusions for this group; nevertheless, due to their apparently increased vulnerability, it is still important to discuss the results for this population.

One of the most relevant explanations for the differential distribution of violence by gender during the migratory process refers to the increasing relations of inequality and pervasive gender roles present within particular migrant groups. This phenomenon may be related to the numerical composition of the migrant flow; men outnumber both women and TTTs, which enables “*machista*” social interactions between migrants, assigning women and TTTs the traditional roles of dependence and subordinance. The perception of transit through Mexico may also contribute to the phenomenon, a country traditionally associated with gender-based inequality and violence [38,39,40,41]. TTTs represent the small minority of the migrant population (less than 1% of the total sample), and are unlikely to obtain social support since they represent stereotypes of individuals whose identities do not conform to the normative male gender standards [42,43]. In this case, hate crimes are exacerbated within migratory contexts, where TTTs represent a minority amidst minorities and usually travel with the exacerbated risks of irregular migratory conditions [44]. In the case of women, traditional gender roles are intensified throughout transit. As shown in this and other similar studies, from the perspective of the male migrant, female migrants require “protection” and are therefore given domestic tasks such as food preparation and laundry in exchange for “protection and security” throughout the journey [38]. Hampered by domestic responsibilities, women are also a focus of harassment and sexual violence. In addition, they are frequently pressured into transactional sex as a means of achieving transit not only for themselves but also for the other members of their migrant groups [45].

Violence against migrants occurs in social contexts of high homicide rates, both in the countries of origin and destination. The countries that form the “Northern Triangle” have the highest homicide rates in Latin America and the Caribbean: El Salvador (60 for every 100,000 inhabitants), Honduras (42.8), and Guatemala (26.1). Mexico, the country of transit for the migrants in question, had a rate of 22.5 homicides for every 100,000 inhabitants in 2017. San Pedro Sula, Honduras, was considered the most violent city in the world in 2013, with a rate of 90.4 homicides for every 100,000 inhabitants [46]. This violent context, present in most countries of origin, is one of the principal motives reported by migrants in their search for a better quality of life. Nevertheless, as the results of this study demonstrate, reports of violence increase over two-fold (2.1) in communities near the US-Mexico border (Saltillo, Coahuila: 38.3%), as compared to the communities in the south where transit begins (Tapachula, Chiapas: 18.2%). This indicates that the more extended the journey, the greater the risk of exposure to violence during the migration process.

### Implications for public policy regarding migration and human rights

The continuously violent context in countries of origin and countries of transit has been reinforced by anti-immigration policies in the US: intensified in the Obama administration and worsened in the current Trump administration with a steeper uptick of persecution and deportation. However, despite these conditions, the majority of migrants who suffer violence in transit decide to continue their journey towards the US.

A periodic update of the migratory context that included our study brings us to lay out the current scene in 2018 and 2019, where new forms of transit through Mexico have been defined: notably, “migrant caravans” [34,47]. Even so, the current government of Mexico (2018–2024) has implemented protective measures for the human rights of transitory migrants, by means of providing a total of 13,270 visitor visas on humanitarian grounds to members of the migrant caravans up to February 11^th^ 2019 [46]. These methods appear to constitute a new social, political, and migratory scene to face the significant challenges related to the prevalent violence in these countries. Migrants are becoming visible, and at the same time, being perceived by the current US government as a threat to national security and has resulted in an emergency response, including militarization at the border, and construction of a wall between the US and Mexico.

### Study limitations

From the start, it is important to note that incidences of violence are largely underreported mainly because victims have normalized and trivialized violence in Central America and Mexico. This may be a reflection of the fact that the interviewees did not consider the majority of their migratory experiences related to violence (i.e., psychological hostility, physical aggressions, and theft) “serious” enough to report. With respect to the way in which violence was categorized, it is important to take into account the difficulty of differentiating between different types of violence, as a result of the complexity of aggressions and the form in which they were reported (self-report).

This study presents limitations related to its cross-sectional design, which limits ability to examine temporal relationships. The study was carried out only with migrants currently occupying the *Casa de migrante*, in order to control the factor of local insecurity and ensure a higher level of participant safety. The process of self-selection of the population which uses the shelters introduces bias in the results obtained, and may affect the generalizability of the findings. However, other studies present a similar prevalence of violence among migrants in general (both users and non-users of shelters) and their sociodemographic characteristics do not differ from the sample considered for this study [27]. Despite its limitations, we believe this study contributes important information and analyses that aid understanding of the role violence plays in the experience of undocumented migrants living in situations of high social vulnerability.

## Conclusions

The results of this paper show that violence is one of the main risks for migrants during their transit through Mexico due to the highly vulnerable conditions they face. In order to understand how violence is exercised, we must fully comprehend its complex dimensions and the social structures that perpetuate it. In this violence-imbued context, migrants in transit are vulnerable to high social risks requiring urgent intervention for the promotion and protection of their rights. The Mexican government has received and committed to comply with a number of recommendations by national and international organizations. However, little has been done to monitor their implementation or outcomes [19,22,47,48,49].

The efforts of the *Casas del migrante*, civil organizations and advocates and defenders of the human rights of migrants are framed in the previously described context of structural violence. Because—or in spite—of this, however, they have come to embody a substantial part of the social response to the consequences of violence against migrants in transit through Mexico. The *Casas del migrante* represent one of the few places where this population can find social support and alleviate some of the negative consequences associated with the violence experienced while migrating. However, shelters do not have the capacity to transform the conditions that determine the magnitude and consequences of violence; this is an issue that must be addressed by the Mexican government and its institutions. To do so, a series of mechanisms should be implemented for effective enforcement of the universal human right to security and social protection. It is necessary to define strategies, which promote and protect the human rights of migrants and secure that they can fully access treatment and care when needed during the entire migration process. The discussion should include and distinguish between the concepts of risks and vulnerability and consider differences based on gender, nationality, ethnicity, and social class among the most relevant for this population. Such response must also address social issues such as stigma, discrimination, and human rights and should be based on the contextual realities. This urgently warrants further investigation aimed at providing evidence for the implementation of the human rights policies necessary to guarantee migrants the effective exercise of their rights.
